# Ascites Volumes and the Ovarian Cancer Microenvironment

**DOI:** 10.3389/fonc.2018.00595

**Published:** 2018-12-17

**Authors:** Marie-France Penet, Balaji Krishnamachary, Flonné B. Wildes, Yelena Mironchik, Chien-Fu Hung, TC Wu, Zaver M. Bhujwalla

**Affiliations:** ^1^Division of Cancer Imaging Research, The Russell H. Morgan Department of Radiology and Radiological Science, The Johns Hopkins University School of Medicine, Baltimore, MD, United States; ^2^Sidney Kimmel Comprehensive Cancer Center, The Johns Hopkins University School of Medicine, Baltimore, MD, United States; ^3^Department of Pathology, The Johns Hopkins University School of Medicine, Baltimore, MD, United States; ^4^Department of Radiation Oncology and Molecular Radiation Sciences, The Johns Hopkins University School of Medicine, Baltimore, MD, United States

**Keywords:** ovarian cancer, ascites, MRI, vascular volume and permeability, total choline

## Abstract

Epithelial ovarian cancer is the leading cause of death from gynecologic malignancy among women in developed countries. Epithelial ovarian cancer has a poor prognosis, due to the aggressive characteristics of the disease combined with the lack of effective therapies. Options for late-stage ovarian cancer are limited and invasive, especially once malignant ascites develops. Malignant ascites, a complication observed in terminal ovarian cancer, significantly contributes to poor quality of life and to mortality. Excess accumulation of fluid in the peritoneal cavity occurs due to a combination of impaired fluid drainage and increased net filtration, mostly due to increasing intraperitoneal vascular permeability. Here we applied non-invasive magnetic resonance imaging (MRI) and spectroscopic imaging (MRSI) of syngeneic mouse tumors *in vivo*, and high-resolution ^1^H MRS of mouse tumor extracts, to characterize the relationship between ascites volumes and the vasculature and metabolism of an experimental model of ovarian cancer. Differences were observed in the tumor vasculature and metabolism in tumors based on ascites volumes that provide new insights into the development of this condition.

## Introduction

Epithelial ovarian cancer is the leading cause of death from gynecologic malignancy among women in developed countries with an estimated incidence of 205,000 cases worldwide per year resulting in ~125,000 deaths (1). Although the prognosis in cases detected at an early stage is quite favorable, the vast majority of cases are diagnosed at a late stage, with a 5 year survival rate below 30% (2, 3). Therapeutic options for advanced stage ovarian cancer are extremely limited and very invasive, especially once malignant ascites develops (2).

Malignant ascites is a complication observed in terminal ovarian cancer that significantly contributes to poor quality of life and to mortality. The excess accumulation of fluid in the peritoneal cavity arises from a combination of impaired fluid drainage and increased net filtration. Malignant ascites formation is thought to occur due to increasing intraperitoneal vascular permeability (4). Local secretion of vascular endothelial growth factor (VEGF) is a key factor in both tumor growth and ascites formation (5). 38% of malignant ascites occurring in women are associated with ovarian cancer. During the course of the disease, more than one-third of women with ovarian cancer will develop ascites (3). Abdominal distension, anorexia, dyspnea, insomnia, fatigue, respiratory distress, low capacity to walk, pain, lower limb discomfort, and edema are among the most common symptoms associated with malignant ascites. Ascites are essentially treated indirectly, using platinum-based intravenous chemotherapy against the underlying disease. When chemoresistant disease has developed, intractable ascites becomes a major problem and the majority of patients receive frequent paracentesis to temporarily alleviate symptoms (3). While repeated ascites drainage can improve the condition, ascites usually recurs in a short period of time. Free-floating cancer cells that are shed from the primary tumor are often present in ascitic fluid, leading to intraperitoneal metastases (4). A majority of women diagnosed with epithelial ovarian cancer have intra-abdominal metastasis at the time of diagnosis. The identification of mechanisms involved in the aggressiveness of ovarian cancers and its associated pathologies, including formation of metastases and build-up of ascitic fluid, is urgently needed to provide new targets for more effective control and treatment.

Non-invasive magnetic resonance imaging (MRI) and magnetic resonance spectroscopic imaging (MRSI) can be used to characterize the tumor microenvironment and understand its role in ascites formation. Here we applied *in vivo* MRI and ^1^H MRSI, and ^1^H MRS of tumor extracts, to better understand the relationship between tumor vasculature, metabolism, and ascites build-up in an experimental model of ovarian cancer. The ID8 cell line is an ovarian epithelial papillary serous adenocarcinoma cell line, originating from mouse ovarian surface epithelial cells transformed after multiple passages *in vitro* (6). The cells were developed to understand early mechanisms in the establishment and progression of ovarian cancer (6). These ID8 cells were further transformed to overexpress VEGF (7). When injected intraperitoneally, ID8-VEGF cells induced multiple tumor nodules localized on the visceral and parietal surfaces of the peritoneal cavity. They are mostly present in the diaphragmatic peritoneum, the porta hepatis, and the pelvis, resembling human ovarian carcinoma (7). These animals also developed ascites that, in late stage of the disease, became hemorrhagic (8). We used the ID8-Defb29 Vegf syngeneic model for our studies. In this model beta-defensin has been added to interact with VEGF-A, and increase tumor vascularization. Beta-defensin and VEGF-A cooperate to promote tumor vasculogenesis. Beta-defensin chemoattracts dendritic cell precursors, whereas VEGF-A primarily induces their endothelial-like differentiation and migration to vessels (9). For most experimental studies of ovarian cancer, ovarian cancer cells are injected into the peritoneal cavity, inducing ascites, and peritoneal spread of tumors. However, with this procedure, most cell lines do not form solid tumors. Instead, here we performed microsurgical orthotopic implantation of ovarian cancer tissue within the ovary of C57BL/6J mice (10). Tumors reached ~300 mm^3^ in 4–6 weeks. After orthotopic tumor implantation, some mice developed high-volume ascites (>50 μl), while others had no or low-volume ascites (< 50 μl). We applied non-invasive MRI and MRSI to better characterize differences in tumor vasculature and metabolism between tumors that produced low and high-volume ascites.

## Materials and Methods

### Cell Lines and Tumor Implantation

ID8-Defb29 Vegf cells were grown in RPMI 1640 medium with 10% fetal bovine serum, and cultured under standard cell culture incubator conditions at 37°C in a humidified atmosphere containing 5% CO_2_. Cells were orthotopically implanted in C57BL/6J mice using a two-step process as previously described (10, 11). First, subcutaneous tumors were generated by inoculating a cell suspension of 2 × 10^6^ ID8-Defb29 Vegf cells in 0.05 ml of Hanks balanced salt solution in the flank of C57BL/6J female mice. Once the tumor reached ~100–200 mm^3^, it was excised, cut into small pieces of comparable sizes under sterilized conditions, and implanted surgically on the ovary of anesthetized C57BL/6J female mice. Mice were scanned every 2 weeks to assess tumor growth. Experiments were performed when orthotopic tumors reached volumes of ~200–300 mm^3^ (corresponding to a diameter of ~7.5–8.5 mm). All surgical procedures and animal handling were performed in accordance with protocols approved by the Johns Hopkins University Institutional Animal Care and Use Committee, and conformed to the Guide for the Care and Use of Laboratory Animals published by the NIH.

### *In vivo* Vascular MRI and MRSI

Imaging studies were performed on a 9.4T Bruker spectrometer (Bruker BioSpin Corp., Billerica, MA) using a 25 mm diameter volume coil placed around the torso of the mouse. Mice were anesthetized with a mixture of ketamine and acepromazine. Anatomic T_1_-weighted images were acquired to localize the orthotopic tumors. To characterize tumor vascular volume and permeability, we used a previously published protocol (12, 13). Briefly, quantitative T_1_ maps were obtained before and after intravenous administration of albumin-GdDTPA (500 mg/kg dose). The albumin-GdDTPA was synthesized as previously described (14). The tail vein of the mouse was catheterized before placing the animal in the spectrometer. Multislice relaxation rate (1/T_1_) maps were obtained by a saturation recovery method combined with fast T_1_ SNAPSHOT-FLASH imaging. First, an M_0_ map with a recovery delay of 10 s was acquired. Then, images of 4 slices (1 mm thick), acquired with an in-plane spatial resolution of 250 μm (128 × 128 matrix, 32 mm FOV, 8 averages) were obtained for three relaxation delays (100 ms, 500 ms, and 1 s). These T_1_ recovery maps were obtained before *i.v*. injection of albumin-GdDTPA and repeated over a 23-min period, starting 3 min after *i.v*. injection of the contrast agent. At the end of the imaging studies, the T_1_ of blood was measured. Relaxation maps were reconstructed from data sets for three different relaxation times and the M_0_ dataset on a pixel-by-pixel basis. Vascular volume (VV) and permeability surface area product (PSP) maps were generated from the ratio of (1/T_1_) values in the images to that of blood.

### Metabolic MRSI

Metabolic maps of tCho were obtained from a 4 mm thick slice using two dimensional-chemical shift imaging (2D-CSI) (15) [echo time (TE) = 135 ms, repetition time (TR) = 1,500 ms, number of acquisition (NA) = 4] with VAPOR water suppression (16). Reference images of the unsuppressed water signal (TE = 20 ms, NA = 2) were acquired to generate quantitative maps in arbitrary units as previously described (17). Processing of the images was done using custom tools developed in Interactive Data Language (IDL).

### Tumors, Ascites, and Metastases

Mice were sacrificed, and the ascitic fluid volume was measured. Lungs, liver, and lymph nodes were excised and fixed in formalin to quantify metastatic spread. Tumors were cut in half with one half freeze clamped for MR extracts and protein analysis, and the other half fixed in formalin.

### MR Spectroscopy of Dual Phase Extracts

Tumor extracts were obtained using a dual-phase extraction method with methanol/chloroform/water (1/1/1) (18, 19). Briefly, tumors were freeze-clamped, ground to powder, and weighed. Ice-cold methanol was added, and the tumor extract samples were homogenized. Chloroform and ice-cold water were finally added. Extract samples were kept at 4°C overnight for phase separation. Samples were then centrifuged for 30 min at 15,000 g at 4°C to separate the phases. The water/methanol phase containing the water-soluble metabolites was treated with chelex (Sigma Chemical Co., St Louis, MO) for 10 min on ice to remove divalent cations. Chelex beads were removed through filtration. Methanol was removed by rotary evaporation, and the remaining water phase was lyophilized. The chloroform from the lipid phase was evaporated using nitrogen gas. Both phases were stored at −20°C until use. Water-soluble extracts were resuspended in 0.6 mL of deuterated water (D_2_O) containing 2.4 × 10^−7^ mol of 3-(trimethylsilyl)propionic 2,2,3,3-d_4_ acid (TSP; Sigma-Aldrich, St. Louis, MO, USA) as an internal standard. Lipid-soluble extracts were resuspended in 0.4 mL of chloroform-D and 0.2 mL of methanol-D4 with 0.05 v/v% tetramethylsilane (TMS) (Cambridge Isotope Laboratories, Inc., Tewksbury, MA, USA) as an internal standard (19). Fully relaxed ^1^H MR spectra of the extracts were acquired on a Bruker Avance 500 spectrometer operating at 11.7 T (Bruker BioSpin Corp., Billerica, MA) using a 5-mm HX inverse probe, and the following acquisition parameters: 30° flip angle, 6,000 Hz sweep width, 9.5 s repetition time, time-domain data points of 32K, and 128 transients (18). Spectra were analyzed using Bruker XWINMR3.5 software (Bruker BioSpin Corp., Billerica, MA). Integrals of the metabolites of interest were determined and normalized to the tumor weight. To determine concentrations, metabolite peak integration values from ^1^H spectra were compared to the internal standards TSP and TMS (19). Statistical significance was evaluated using the Student *t*-test. *P*-values ≤ 0.05 were considered statistically significant.

### Immunoblot of Cells and Tumors Extracts

Proteins were extracted from freeze-clamped tumors using radioimmunoprecipitation lysis buffer fortified with a protease inhibitor cocktail, dithiothreitol, phenylmethylsulfonyl fluoride, sodium orthovanadate, and sodium fluoride (Sigma Chemical Co., St Louis, MO). Protein concentration was estimated using the Bradford Bio-Rad protein assay kit (Bio-Rad, Hercules, CA). About 60 μg of total protein was resolved on 7.5% SDS-PAGE gels from Bio-Rad, transferred onto nitrocellulose membranes, and probed with antibodies directed against mouse FAS (A-5) (Santa Cruz Biotechnology; dilution 1:400), cPLA2 (Santa Cruz Biotechnology; dilution 1:200), ApoE (M-20) (Santa Cruz Biotechnology; dilution 1:200). GAPDH was used as a loading control and detected with a monoclonal antibody (Sigma Aldrich, dilution 1:50,000). Immunoblots were developed using SuperSignal West Pico chemiluminescent substrate kit (Thermo Scientific, Rockford, IL).

## Results

**Figure 1:**
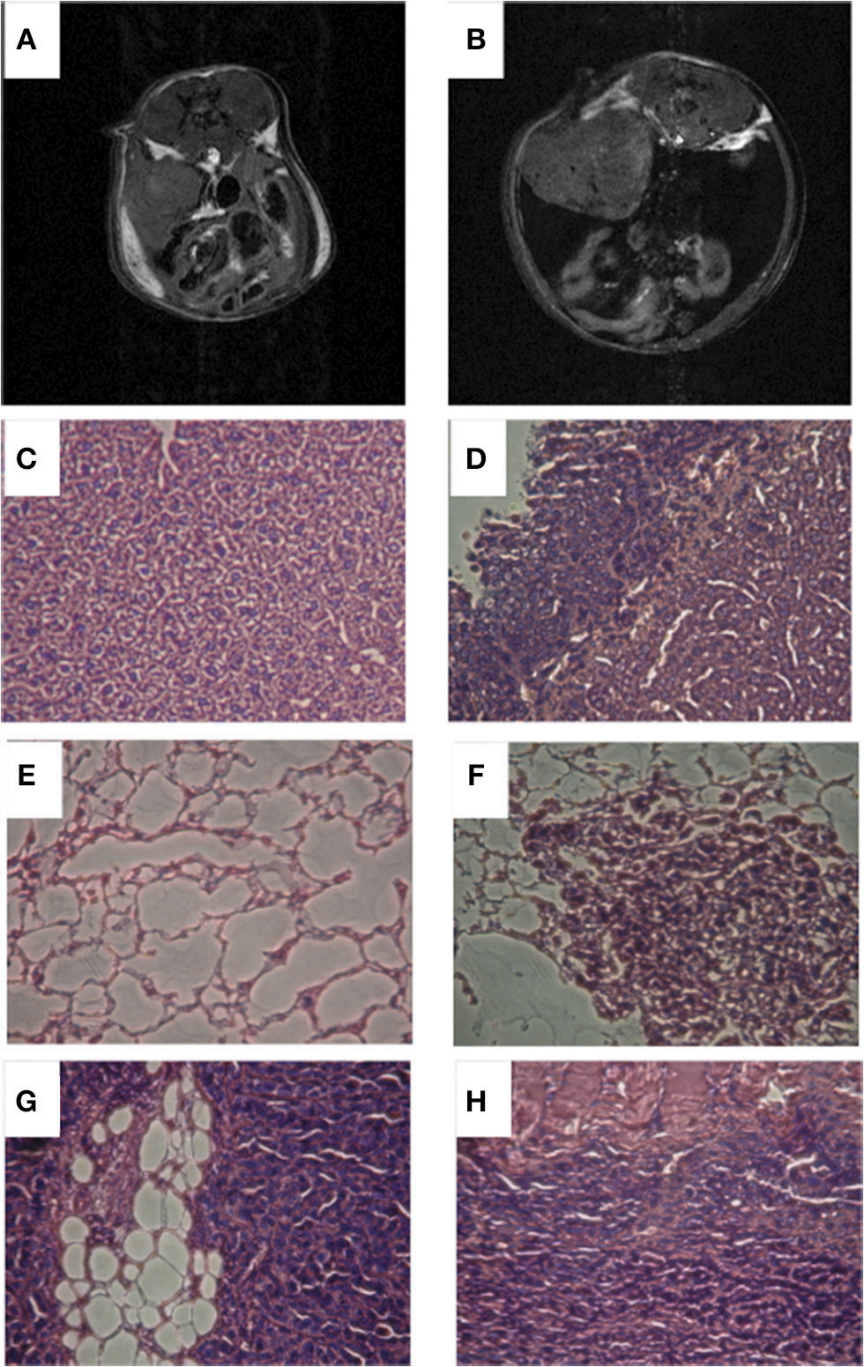
Representative anatomic T_1_ weighted images of a mouse with no ascites **(A)** and a mouse with high ascites **(B)**. Representative histological images of liver from mouse with no ascites **(C)**, liver from mouse with high ascites **(D)**, lungs from mouse with no ascites **(E)**, lungs from mouse with high ascites **(F)**, intestine from mouse with high ascites **(G)**, diaphragm from mouse with high ascites **(H)**.

Mice were imaged when tumors were ~200–300 mm^3^. As shown in Figures 1A,B, two groups were identified based on no or low-volume (Figure 1A) and high-volume (Figure 1B) ascites. Ascitic fluid detected in the MR anatomic images, was characterized by the presence of a dilated abdomen and low intensity signal present inside the peritoneal cavity. The presence or absence of ascites was confirmed *ex vivo*. Metastases were more frequent in mice with ascites, especially in organs in the peritoneal cavity, including the diaphragm (67 *vs*. 0%), liver (100 *vs*. 20%) and intestine (17 *vs*. 0%), as shown in Figures 1C–H. Metastases in the lungs were observed in 67% of mice with high ascites compared to 60% in the mice with no ascites.

**Figure 2:**
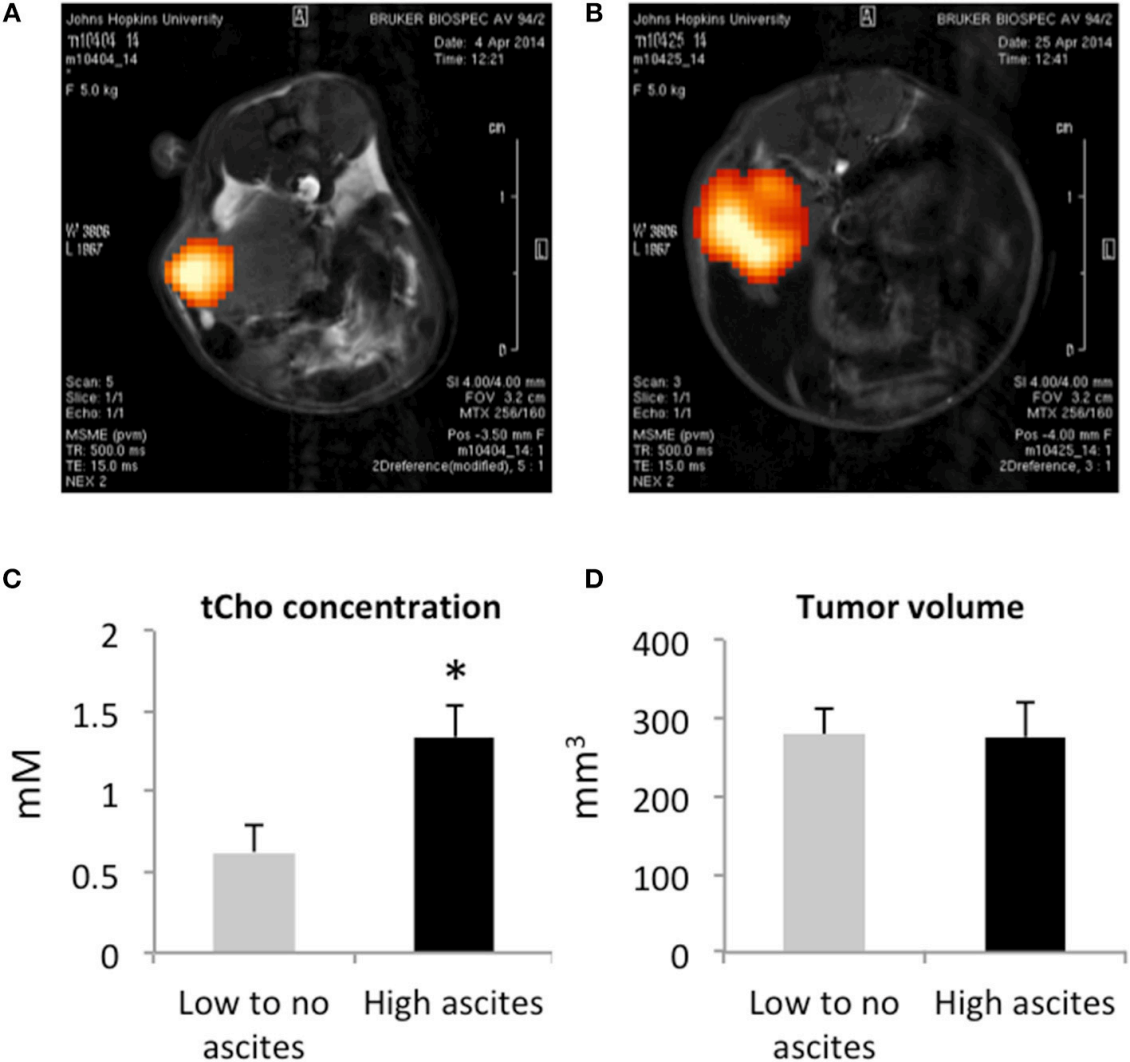
Representative tCho density maps in a mouse with no ascites **(A)** and in a mouse with high ascites **(B)**. Tumor tCho concentrations in mice with no to low-volume ascites and in mice with high-volume ascites **(C)** (*n* = 5 and *n* = 7, respectively; *p < 0.05). Tumor volume in mice with no to low-volume ascites and in mice with high-volume ascites **(D)** (*n* = 5).

Total choline (tCho) was detected with ^1^H MRSI in all the orthotopic tumors imaged (Figures 2A,B). The tCho signal represents the sum of free choline, phosphocholine (PC), and glycerophosphocholine (GPC) that appears as a single peak in ^1^H MR spectra acquired *in vivo*. As shown in the representative images, the signal was heterogeneous in the tumors, confirming the importance of acquiring ^1^H MRSI rather than single voxel MRS when possible. We quantified the tCho signal and observed a significantly higher concentration of tCho in the mice presenting with high-volume ascites (Figure 2C). There was no difference in tumor volumes between the two groups (Figure 2D) suggesting that, in this model, ascites build up was independent of tumor size. There was no correlation between the amount of ascites formed and the duration of tumor progression.

**Figure 3:**
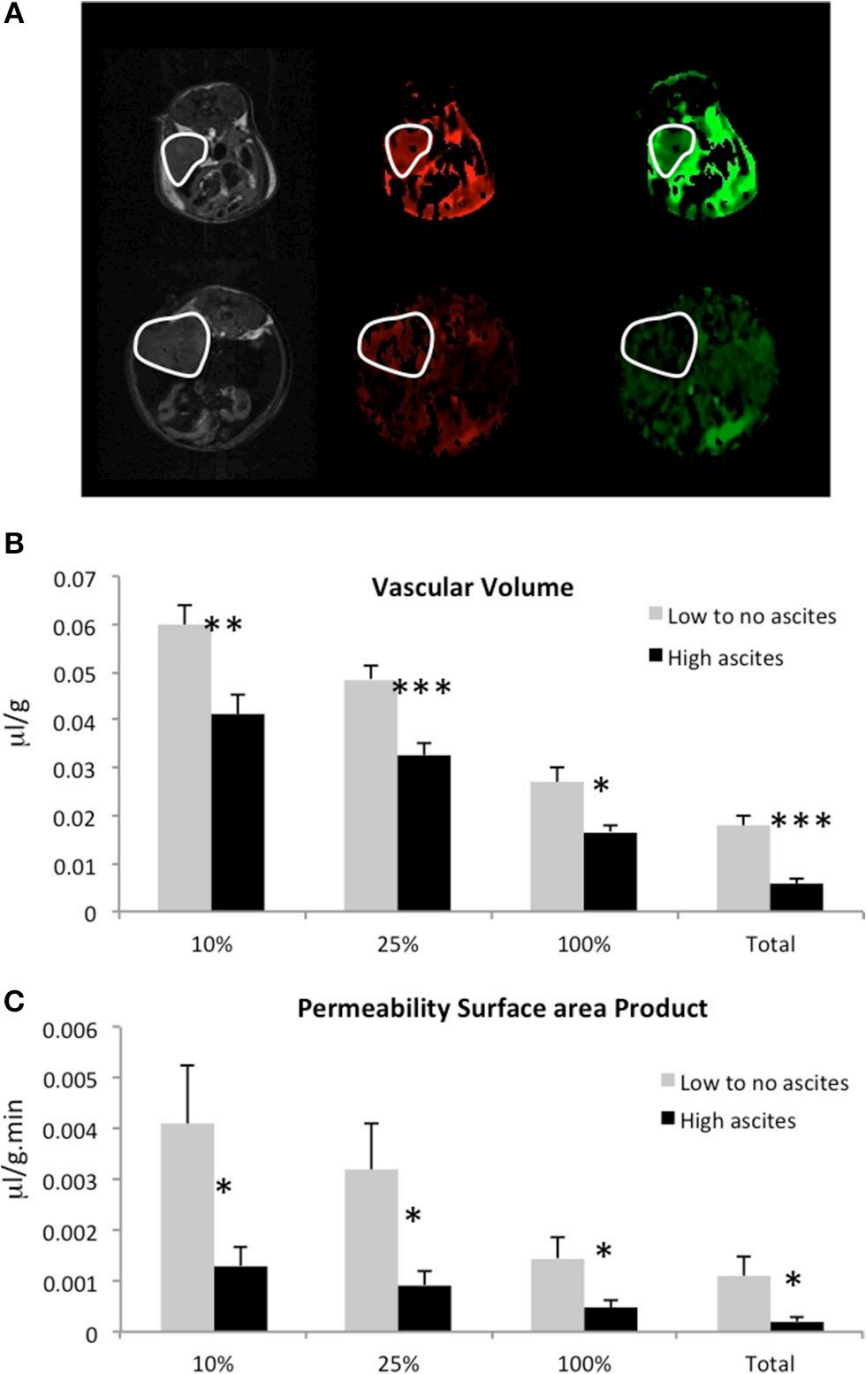
**(A)** Representative anatomical images, vascular volume maps, and permeability surface area product maps in a mouse with no ascites (top row) and in a mouse with high-volume ascites (bottom row). Tumors are highlighted in white. Vascular volume **(B)** and permeability surface area product **(C)** values for the highest 10, 25, 100% non-zero values, and for the total voxels are shown here (*n* = 5; *p < 0.05, **p < 0.01, ***p < 0.005).

We measured vascular volume (VV) and permeability surface area product (PSP) in the ID8-Defb29 Vegf tumors using MRI of the macromolecular contrast agent albumin-gadolinium-DTPA. Representative maps are shown in Figure 3A, in a mouse without ascites (top row), and in a mouse with elevated amount of ascites (bottom row). Quantification of these maps revealed significantly lower VV and lower PSP in the tumors from the mice with high-volume ascites compared to low-volume ascites (Figures 3B,C).

**Figure 4:**
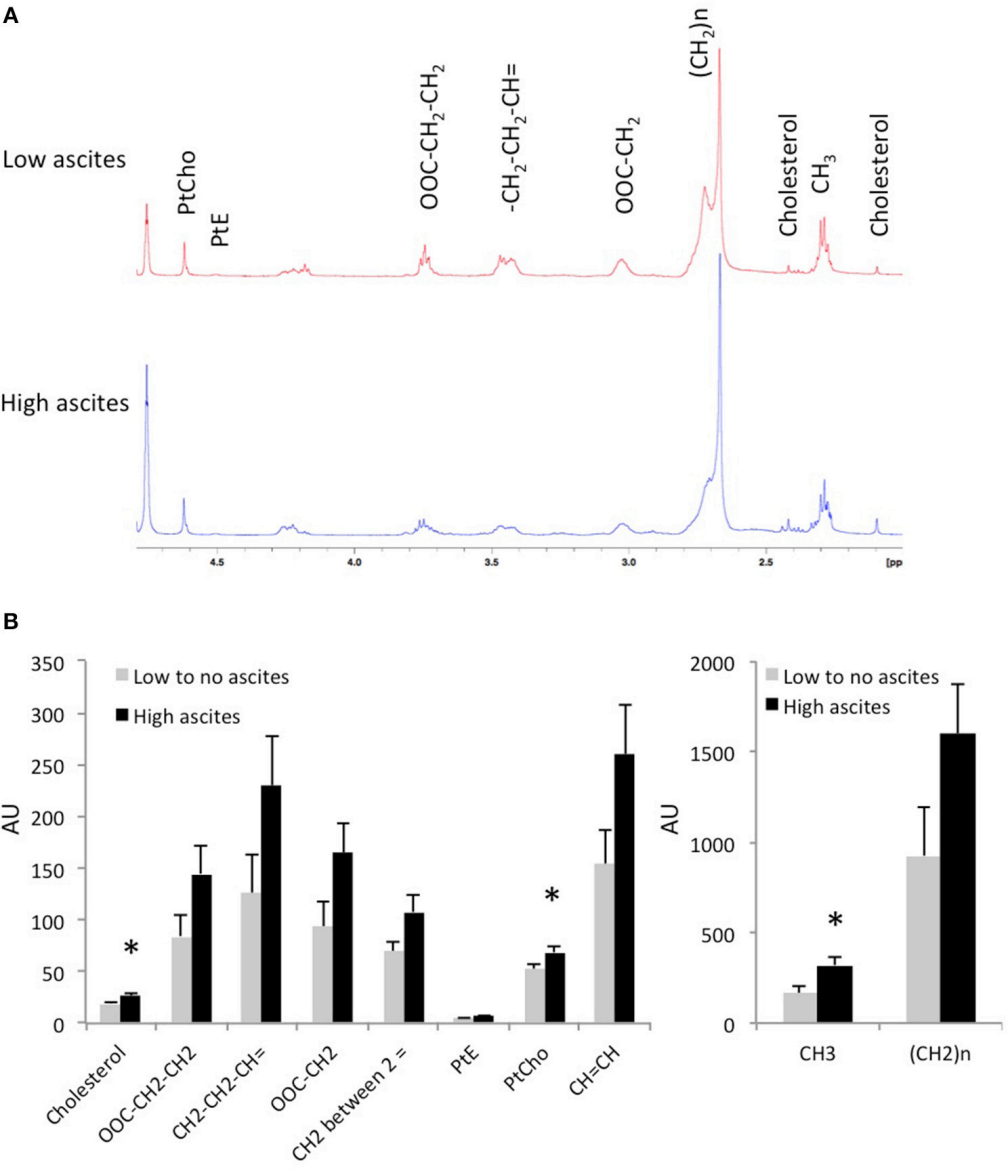
**(A)** Representative tumor lipid ^1^H MR spectra from a mouse without ascites and a mouse with high-volume ascites are shown here. **(B)** Lipids concentration in tumor extracts from mice with no to low-volume ascites and mice with high-volume ascites in arbitrary units (*n* = 6; *p < 0.05).

To further examine the metabolic differences between both tumor types, we extracted the tumors and performed high resolution ^1^H MRS. Analysis of the lipid phase obtained after dual phase extraction revealed higher concentrations of cholesterol, phosphatidylcholine (PtdCho), phosphatidylethanolamine (PtdE), and lower CH_2_/CH3 ratio in tumors from mice with high-volume ascites (Figure 4). Figure 4A shows representative lipid phase ^1^H MR spectra. Quantification of the data is shown in Figure 4B (*n* = 6, *p* < 0.05). No significant differences were observed in the water phase tumor extracts (data not shown).

**Figure 5:**
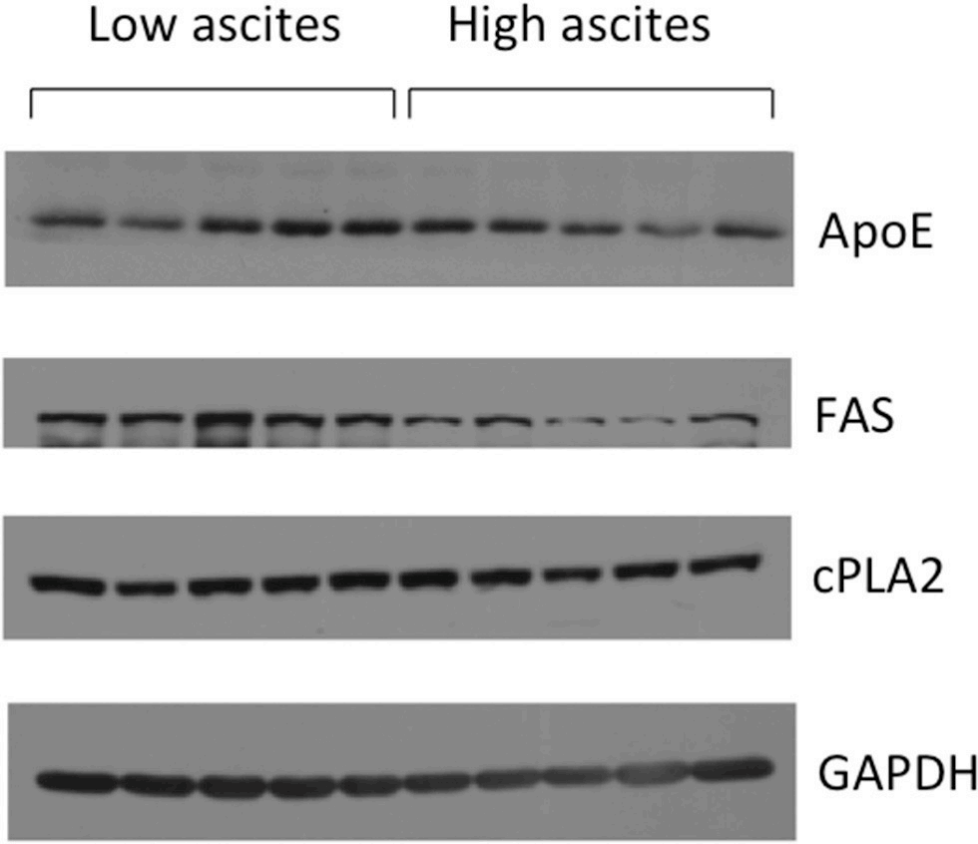
Representative immunoblots showing cPLA2, ApoE, and FAS expression levels in mice with low ascites (*n* = 5) and mice with high ascites (*n* = 5). GAPDH was used as loading control.

To better understand the differences observed in tumor lipid patterns, we investigated the expression levels of some of the proteins involved in lipid and cholesterol metabolism (Figure 5). While no significant differences were observed in the expression of cytosolic phospholipase A2 (cPLA2) and ApoE, we observed a lower expression of fatty acid synthase (FAS) in tumors from the high-volume ascites group.

We also performed qRT-PCR to analyze tumor Chk and VEGF mRNA expression levels and found no significant differences between the no or low-volume and high-volume ascites groups (data not shown). The differences in ascites volume, and in VV and PSP measured *in vivo* between the groups do not appear to be directly related to VEGF expression in the tumors.

## Discussion

Despite a similar genetic background, C57BL/6J mice implanted with ID8-Defb29 Vegf tumors did not develop similar volumes of ascites. While some developed high-volume ascites, others presented with low-volume or no ascites. Mice with high-volume ascites presented with more metastases, especially on the diaphragm, intestine, and liver, confirming the role of ascitic fluid in ovarian cancer cell dissemination and metastatic spread. In ovarian cancer, metastases can occur through different routes. Malignant cells can shed directly into the peritoneal cavity from the primary tumor, disseminate within the cavity, and are carried by the peritoneal fluid to the peritoneum, diaphragm, and omentum. Cells can also disseminate *via* the lymphatic system, resulting in a high rate of pelvic and para-aortic lymph node involvement. Hematogenous spread is less predominant but can also occur in ovarian cancer. In a retrospective clinical study, 372 ovarian cancer patients were divided into 2 groups, depending of the presence or absence of ascites (20). There were no differences observed in tumor size and disease stage, similarly to what we observed in our study. However, a correlation between the presence of ascites and intraperitoneal and retroperitoneal tumor spread was observed (20). Moreover, presence and volume of ascites were significantly related to patient survival (20).

We observed significantly lower VV and PSP in tumors of mice with high-volume ascites. Transendothelial leakage of proteins and macromolecules from microvessels into the peritoneal cavity are linked to ascites formation, by passively drawing water into the peritoneum due to an osmotic effect (21). VEGF and the resultant increased vessel permeability have been implicated in ascites formation (5, 21, 22). Anti-VEGF therapy in a rat model of ovarian cancer resulted in a decrease of ascites formation and an MRI-detected decrease of vascular permeability (21). Under the influence of increased VEGF, tumor microvessels become hyperpermeable. The relationship between ascites volumes and vessel permeability has not been directly evaluated. The significant decrease of VV and PSP observed in the high-volume ascites group may be due to the pressure created by the high volume of ascites in the peritoneal cavity causing a collapse of vessels that blocked albumin-Gd-DTPA delivery and limited its extravasation. In our study, all tumors overexpressed VEGF. As a result, we did not observe any differences in VEGF expression levels. However, all the mice did not form similar volumes of ascites implicating additional factors in regulating ascites volumes. Our results imply that high-volume ascites are likely to result in tumors with poor vascular delivery that can have major consequences for treatment delivery and therapeutic efficacy in ovarian cancer patients.

Several studies have also demonstrated the role of lymphatic obstruction in tumor related ascites (23). Cells, proteins, and macromolecules are preferentially located within the intravascular space. However, they can also leak and accumulate in the peritoneal cavity, and return into the systemic circulation through the peritoneal lymphatic system. It is possible that lymphatic drainage was more effective in mice with no or low-volume ascites.

Differences were also observed in tumor metabolism between the two groups with higher concentrations of cholesterol, PtCho, and PtE in the tumors of mice characterized by high-volume ascites. Lipids play a critical role in cell growth, division, and apoptosis regulation, serving as a chemical energy storage source, cell membrane structural components, and signal transduction molecules. Lipids can be broken down into bioreactive lipid mediators that regulate multiple carcinogenic processes including cell growth, cell migration, and metastasis formation (24). Human ovarian cancer ascites and blood have been shown to contain high level of biologically active lipid factors (25). Bioactive lipid factors, such as lysophosphatidic acid (LPA), can be produced by peritoneal mesothelial cells and by ovarian cancer cells. LPA increases cancer cell migration, cell invasion through peritoneal mesothelial cell monolayers, and cell adhesion to collagen 1 fibers, all of which are necessary for metastasis formation. Ovarian cancer cells are characterized by a hyperactive lipogenesis, with elevated *de novo* lipid synthesis rate (26). FAS is a key enzyme in these processes (26, 27). Increased FAS has been previously observed in ovarian cancer (26, 27). While we did measure FAS in both groups of mice, our data showed a lower FAS expression in mice with high volume ascites.

Phospholipase A_2_ (PLA_2_) plays a critical role in ovarian tumor progression and ascites formation (28). This enzyme breaks down PtCho, the major membrane lipid, to form lyso-phosphatidylcholine (lyso-PtdCho). cPLA_2_ activity is higher in epithelial ovarian cancer tissues compared to benign or normal tissues (28), and higher levels of lyso-PtdCho and arachidonic acid are measured in epithelial ovarian cancer ascites, compared to benign liver cirrhosis (28, 29). Here, we did not observe any differences in cPLA2 expression levels between both groups, despite the higher level of PtdCho in the tumors of the mice presenting with high volume ascites. Although there was no difference in the cPLA2 levels in the tumor obtained from both high and low ascites volumes, it is possible that other subtypes of PLA2 like calcium independent iPLA2 or sPLA2 may be differentially expressed in these tumor types (28).

Higher levels of cholesterol were measured in the tumors that presented with high-volume ascites. High levels of cholesterol have been previously detected in aggressive mouse ovarian surface epithelial cells (30). The elevated concentration of cholesterol in ovarian tumors can be explained by an increased uptake of low-density lipoprotein (LDL) that contains most of the cholesterol in the plasma (31). Cancer cell proliferation requires increased energy metabolism and membrane biosynthesis that could explain the uptake of lipoprotein observed in malignant ovarian cancers, along with an upregulation of the LDL receptor (31). It has been shown that ovarian cancer cells exposed to the ovarian microenvironment have increased expression of multiple enzymes involved in the mevalonate pathway, the first metabolic steps of cholesterol synthesis (32). Moreover, Simvastatin, an inhibitor of HMG-CoA reductase, key enzyme involved in cholesterol biosynthesis, can inhibit the growth of ID8 ovarian lesions (32). Apolipoproteins are multifunctional proteins transporting cholesterol, triglycerides, and phospholipids in circulatory fluids. Their metabolism and biosynthesis are dysregulated in malignant ovarian tumors. ApoE, an essential constituent of plasma lipoproteins, is overexpressed in ovarian cancer (33). Responsible for cholesterol metabolism and transport, it plays a critical role in proliferation and survival of ApoE-expressing ovarian cancer cells (34). The differences we observed in cholesterol levels were, however, not due to differences in ApoE expression.

## Conclusion

In summary, despite similar genetic backgrounds and VEGF expression levels, the ID8-Defb29 Vegf tumors implanted orthotopically induced very different ascites volumes, highlighting the importance of tumor microenvironmental factors in the accumulation of ascites. Our data suggest that large volumes of ascites may act to occlude vessels affecting delivery of therapeutic agents. These studies provide new insights into vascular and metabolic differences in no or low-volume ascites and high-volume ascites that merit expanded future investigation.

## Author Contributions

M-FP and ZB contributed to the conception and design of the study. M-FP, BK, YM, FW acquired the data. M-FP performed the data analysis and wrote the first draft of the manuscript. BK, YM, FW, TW, C-FH, and ZB revised the manuscript critically. All authors contributed to manuscript revision, read and approved the submitted version.

### Conflict of Interest Statement

The authors declare that the research was conducted in the absence of any commercial or financial relationships that could be construed as a potential conflict of interest.

## Abbreviations

Chk: choline kinase
Cho: free choline
CSI: chemical shift imaging
FAS: fatty acid synthase
GPC: glycerophosphocholine
MRI: magnetic resonance imaging
MRS: magnetic resonance spectroscopy
MRSI: magnetic resonance spectroscopic imaging
PC: phosphocholine
PtdCho: phosphatidylcholine
PtdE: phosphatidylethanolamine
tCho: total choline
VEGF: vascular endothelial growth factor.
